# RURAL–URBAN DISPARITIES IN COGNITIVE PERFORMANCE IN BRAZIL AND MEXICO

**DOI:** 10.1093/geroni/igad104.0946

**Published:** 2023-12-21

**Authors:** Natalia Gomes Goncalves, Jaqueline Avila, Laiss Bertola, Alejandra Obregon, Cleusa Ferri, Rebeca Wong, Claudia Suemoto

**Affiliations:** University of Sao Paulo Medical School, Sao Paulo, Sao Paulo, Brazil; University of Massachusetts Boston, Boston, Massachusetts, United States; Federal University of Sao Paulo, Sao Paulo, Sao Paulo, Brazil; University of Texas Medical Branch, Galveston, Texas, United States; Federal University of Sao Paulo, Sao Paulo, Sao Paulo, Brazil; UTMB, Galveston, Texas, United States; Universidade de São Paulo, São Paulo, Sao Paulo, Brazil

## Abstract

Latin America (LA) has the highest dementia burden globally among people 60 years or older, with a prevalence of 8.5%. However, there is little evidence of the disparities in cognitive performance in rural and urban areas in LA. We use nationally representative data from the two largest countries in LA, Brazil and Mexico, to assess if rurality is associated with cognitive impairment, and assess how education and country modify this relationship. The sample included 9,412 participants from the Brazilian Longitudinal Study of Aging (ELSI) and 14,779 participants from the Mexican Health and Aging Study (MHAS). Participants were classified as cognitively impaired or not impaired using cognitive performance scores and a regression-based approach. Logistic regression models were used to estimate the association between rural/urban residency and cognitive function. The modifying effect of education and country was investigated by adding an interaction term in the fully adjusted model. ELSI participants had a mean age 62.5±9.8 years, 54% were women, 13.3% were illiterate, and 6% had cognitive impairment. MHAS participants from had a mean age 64.8±9.7 years, 55% were women, 18% were illiterate, and 5% were cognitively impaired. In Mexico, those living in rural areas were more likely to have cognitive impairment than those in urban areas (OR=1.43, 95%CI=1.20;1.71), but this difference was not seen in Brazil (OR=1.10, 95%CI=0.87;1.36). The three-way interaction of rurality with country and education showed that rural residents of Mexico had lower odds of cognitive impairment compared to rural residents of Brazil with the same level of education (p<0.001).

